# Primary Anaplastic Lymphoma Kinase‐Positive Inflammatory Myofibroblastic Tumor of the Small Bowel Detected by Capsule Endoscopy: A Case Report

**DOI:** 10.1002/deo2.70327

**Published:** 2026-04-11

**Authors:** Tomoyuki Niwa, Yasushi Hamaya, Yusuke Asai, Tatsuhiro Ito, Satoru Takahashi, Shunya Onoue, Satoshi Osawa, Mayu Sakata, Hiroya Takeuchi, Ken Sugimoto

**Affiliations:** ^1^ Department of Endoscopic and Photodynamic Medicine Hamamatsu University School of Medicine Shizuoka Japan; ^2^ First Department of Medicine Hamamatsu University School of Medicine Shizuoka Japan; ^3^ Department of Advanced Medical Science for Regional Collaboration Hamamatsu University School of Medicine Shizuoka Japan; ^4^ Second Department of Surgery Hamamatsu University School of Medicine Shizuoka Japan

**Keywords:** anaplastic lymphoma kinase, capsule endoscopy, inflammatory myofibroblastic tumor, laparoscopy, small intestine

## Abstract

Primary inflammatory myofibroblastic tumor (IMT) of the small intestine is rare, and its endoscopic characteristics remain poorly defined. We report a case of anaplastic lymphoma kinase (ALK)‐positive small bowel IMT detected by capsule endoscopy (CE). A 24‐year‐old woman presented with epigastric pain. Contrast‐enhanced computed tomography revealed a 2‐cm mass in the small intestine. Double‐balloon endoscopy was attempted but failed to reach the lesion. CE demonstrated a submucosal tumor‐like protrusion with mild surface erythema and conspicuous whitish villous changes. Laparoscopy‐assisted partial resection of the small intestine was performed, and histopathological examination with immunohistochemistry confirmed ALK‐positive IMT. The postoperative course was uneventful, and no recurrence was observed during 8 months of follow‐up. This case indicates that CE can provide clinically useful information for the evaluation of small bowel submucosal lesions beyond the reach of conventional endoscopy. IMT should be considered in the differential diagnosis of erythematous small bowel tumors in young patients.

## Graphical abstract

We present a 24‐year‐old female with epigastric pain diagnosed with a primary anaplastic lymphoma kinase‐positive inflammatory myofibroblastic tumor (IMT) of the small bowel. When double‐balloon enteroscopy failed to reach the lesion, capsule endoscopy (CE) successfully identified an erythematous submucosal tumor with whitish villi, leading to surgical resection. This case highlights CE as a vital rescue diagnostic modality and suggests IMT should be considered for small bowel tumors presenting with these specific endoscopic features.

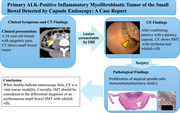

## 1 Introduction

Inflammatory myofibroblastic tumor (IMT) is a mesenchymal tumor characterized by myofibroblastic spindle‐cell proliferation with inflammatory cell infiltration [1]. The World Health Organization classifies IMT as an intermediate‐grade tumor due to recurrence and rare metastatic potential [2]. Although IMT most commonly arises in the lungs, extrapulmonary involvement, including the abdominal cavity, has been reported. Primary IMT of the small intestine is extremely rare [3].

Preoperative diagnosis is challenging because radiologic findings are nonspecific and endoscopic access is limited. Descriptions of endoscopic findings of small bowel IMT are scarce, and reports documenting capsule endoscopy (CE) findings are particularly rare. We report a case of primary small bowel anaplastic lymphoma kinase (ALK)‐positive IMT with characteristic CE findings and discuss the endoscopic features and diagnostic implications.

## 2 Case Report

A 24‐year‐old woman presented with epigastric pain. Her medical history was notable only for dysmenorrhea. Computed tomography (CT) performed at a local clinic revealed a small bowel mass with proximal dilation, and she was referred to a general hospital. By the time she presented to our hospital, her symptoms had resolved, and repeat contrast‐enhanced CT showed resolution of the bowel dilation. She was subsequently referred to our department for further evaluation.

**FIGURE 1:**
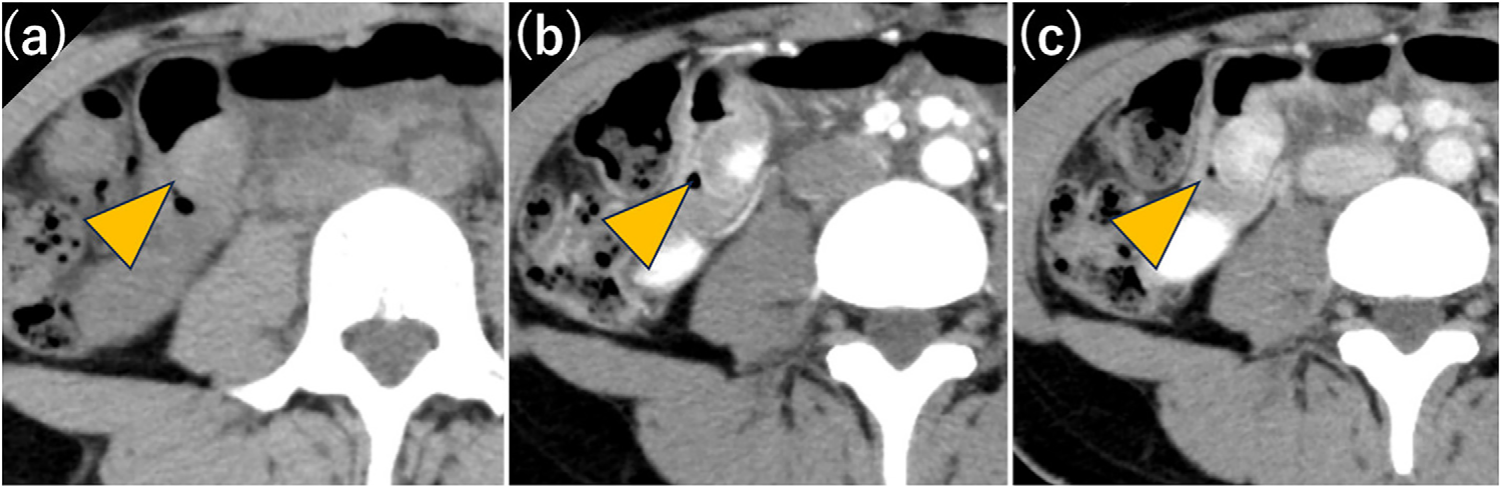
Abdominal computed tomography (CT) findings. (a) Plain CT. (b) Early‐phase contrast‐enhanced CT. (c) Portal venous phase. The tumor (arrowhead) is recognized as a smooth, elevated lesion within the small intestine, showing progressive enhancement.

**FIGURE 2:**
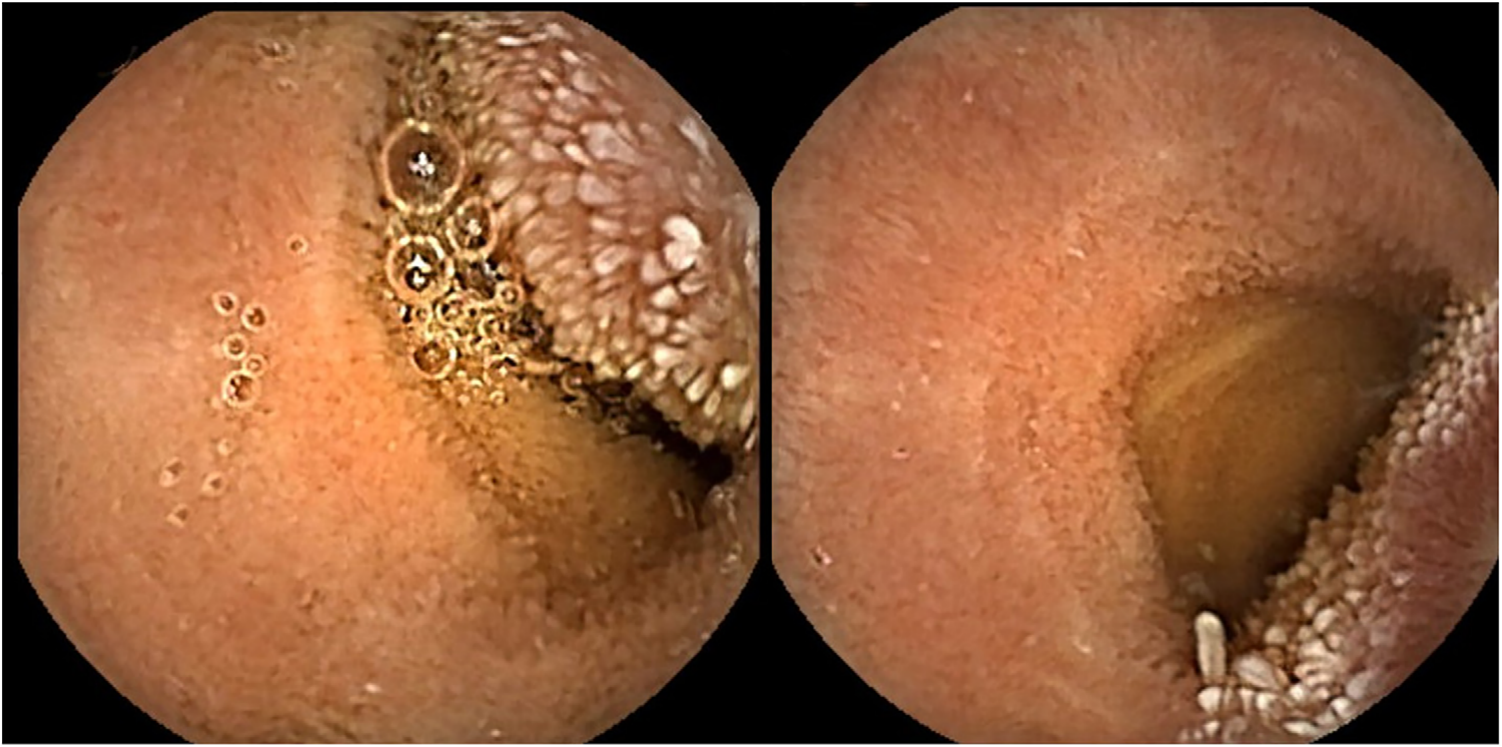
Capsule endoscopy findings. The tumor was visualized 2 h and 18 min after ingestion as a mildly erythematous submucosal tumor (SMT)‐like lesion accompanied by whitish villi. No capsule retention was observed.

**FIGURE 3:**
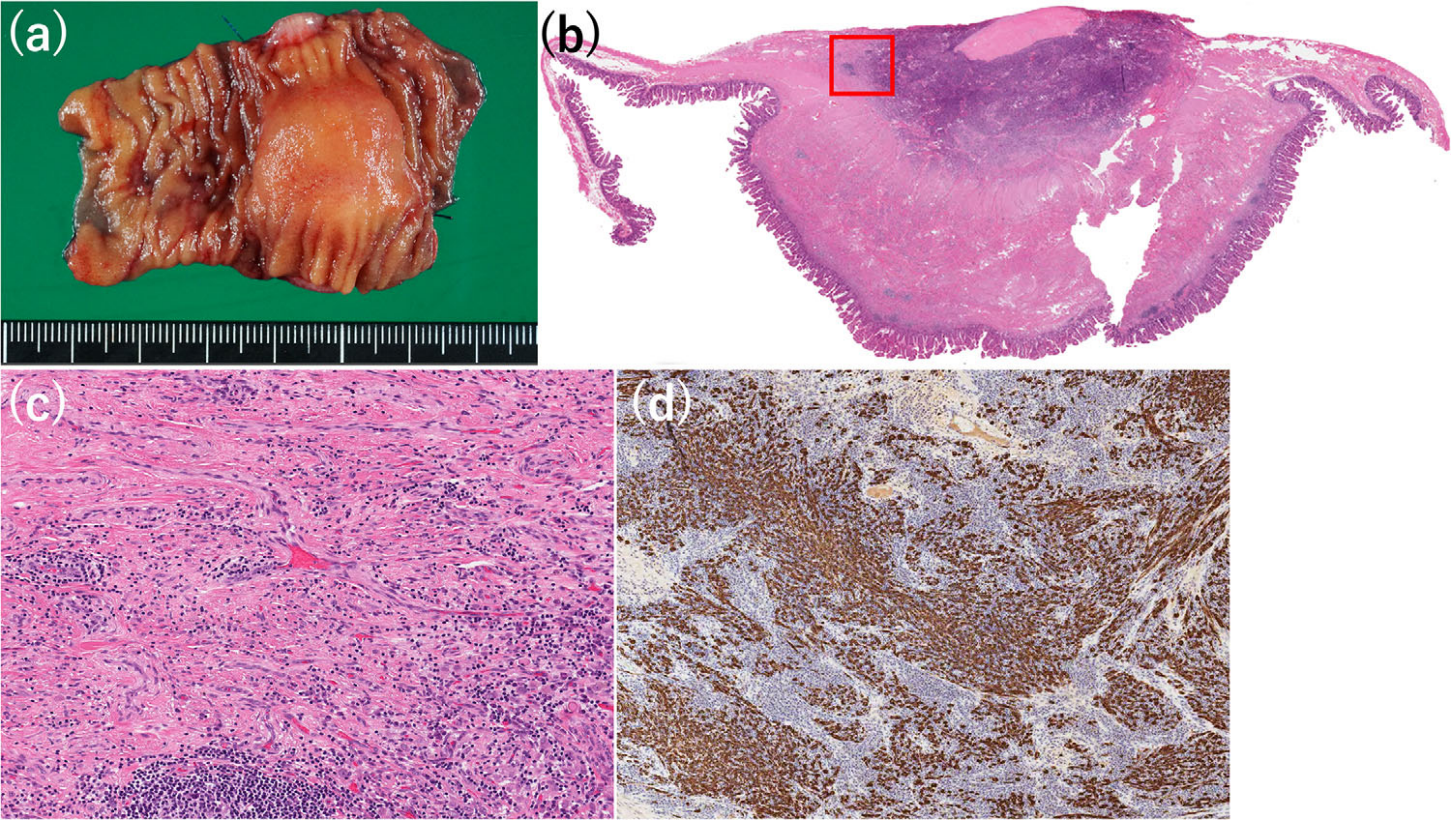
Macroscopic and histopathological findings. (a) Macroscopic view showing a 22 × 20 × 15 mm submucosal tumor. (b) H&E staining showing an ill‐defined lesion extending from the deep mucosa to the serosa with collagen fiber proliferation and inflammatory cell infiltration. (c) High‐power view showing fascicular proliferation of atypical spindle‐shaped to irregularly rounded cells. (d) Immunohistochemistry showing anaplastic lymphoma kinase (ALK) positivity.

On admission, physical examination and laboratory tests (including inflammatory and tumor markers) were unremarkable. Abdominal ultrasonography revealed a heterogeneous hypoechoic mass with abundant intralesional blood flow. Contrast‐enhanced CT demonstrated a 2‐cm mass in the small intestine that appeared mildly hyperdense on plain images and showed gradual enhancement from the center toward the periphery on dynamic contrast phases (Figure 1). Although CT showed no obstruction, localized wall thickening suggested a transient obstructive event. Double‐balloon endoscopy was attempted via both oral and anal approaches; however, despite the administration of adequate sedation and analgesia, the procedure had to be discontinued prematurely due to severe patient pain and intolerance. Consequently, the lesion could not be reached. An intraprocedural Gastrografin study also failed to identify it. Because the suspected lesion was relatively small on CT, despite the transient obstructive episode, we pursued further evaluation. To safely mitigate capsule retention risk, a patency capsule was administered; following uneventful excretion, CE was performed. CE revealed a submucosal tumor (SMT)‐like protrusion 2 h and 18 min after ingestion, exhibiting mild erythema and conspicuous whitish villi, presumed to represent lymphatic congestion (Figure 2). Capsule passage was uneventful. Differential diagnosis based on CE included benign lesions (e.g., leiomyoma) and malignant SMTs (e.g., gastrointestinal stromal tumor, neuroendocrine tumor, and lymphoma). Because standard endoscopic biopsy of SMTs is diagnostically challenging, and given the patient's obstructive history, surgical resection was selected over repeat endoscopy. Laparoscopy‐assisted partial resection of the small intestine was performed. Although preoperative endoscopic marking was not achieved, comparing the sequential CT scans revealed tumor mobility, suggesting it could be readily located intraoperatively. During laparoscopy‐assisted surgery, systematic inspection from the terminal ileum successfully identified the tumor 100 cm proximally as a whitish serosal lesion. After confirming the absence of other lesions, the involved segment was exteriorized through the umbilical port, palpated directly, and segmentally resected with regional lymph node dissection. Gross examination revealed a 22 × 20 × 15 mm submucosal mass with a whitish cut surface and focal discoloration. Histologically, the lesion extended from the deep mucosa to the serosa and consisted of fascicular proliferation of atypical spindle‐shaped to irregularly rounded cells within a background of collagen deposition and dense inflammatory infiltration. Additionally, localized lymphatic dilation was sporadically observed in the overlying and surrounding mucosa, although severe edema was absent. Immunohistochemistry showed cytoplasmic positivity for ALK, partial positivity for α‐smooth muscle actin, and diffuse positivity for desmin, while staining for S‐100, CD34, and DOG‐1 was negative. The Ki‐67 labeling index was approximately 10%. Surgical margins were negative, and no lymph node metastasis was identified. These findings established the diagnosis of ALK‐positive IMT (Figure 3).

The postoperative course was uneventful, and the patient was discharged on postoperative day 6. She has remained free of recurrence for 8 months.

## 3 Discussion

Small bowel IMT is rare, and preoperative diagnosis remains difficult. Although IMT was previously considered a reactive inflammatory condition [4], increasing evidence supports its neoplastic nature in a substantial proportion of cases. Chromosomal abnormalities involving the 2p23 region harboring the *ALK* gene have been identified in approximately half of IMTs, and aberrant ALK protein expression is reported in 50%–60% of cases [5]. In the present case, there was no history of trauma or surgery, and immunohistochemistry demonstrated cytoplasmic ALK positivity, supporting the diagnosis of ALK‐positive IMT.

IMT can arise in soft tissues throughout the body, including the abdominal cavity, retroperitoneum, and pelvic organs, in addition to the lungs [3]. Abdominal IMTs most commonly affect the liver and mesentery, with the small intestine involved in only 15.4% of cases [6].

We searched PubMed for English‐language case reports (2015–2025) using the terms “inflammatory myofibroblastic tumor” AND “small intestine”. Defining “primary small bowel IMT” strictly as tumors originating within the small bowel wall (excluding direct extensions), we summarized 17 cases detailing their clinical characteristics and publication identifiers (Table 1). Most patients were relatively young and presented with obstructive symptoms, and surgical resection was performed in all cases. Preoperative diagnosis was rarely achieved, and ALK positivity was observed in a subset of cases. Notably, the tumor in our case was relatively small compared with previously reported lesions.

**TABLE 1:** Clinical characteristics of reported primary small bowel inflammatory myofibroblastic tumor (IMT) cases (*n* = 17).

	Author, Year	Age/Sex	Size (cm)	Location	Treatment	DOI
1	Xing et al., 2025	7/F	3.0 × 4.0	Ileum	Surgery	10.3389/fonc.2025.1512402
2	Maqbool, 2024	29/M	6.0 × 5.0	Mid small intestine	Surgery	10.1016/j.ijscr.2024.110438
3	Dinçer et al., 2024	46/M	6.0 × 1.5	Terminal ileum	Surgery	10.14744/tjtes.2024.82091
4	Asbah et al., 2023	2/M	15.0 ×12.0	Small intestine	Surgery	10.1016/j.ijscr.2023.108871
5	Moges et al., 2023	55/M	3.0 × 4.0	Jejunum	Surgery	10.1016/j.ijscr.2023.108404
6	Kaspar et al., 2023	60/F	6.5	Distal jejunum, Proximal ileum	Surgery	10.7759/cureus.36798
7	Budylev et al., 2022	23/F	4.4	Terminal ileum	Surgery	10.3941/jrcr.v16i1.3928
8	Farhan, 2020	23/F	3.0 × 3.0	Mid ileum	Surgery	10.7759/cureus.10902
9	Alabbas et al., 2020	7/M	2.5 × 2.0	Ileum	Surgery	10.1093/jscr/rjaa322
10	Hirose et al., 2020	62/M	4.3 × 2.3	Terminal ileum	Surgery	10.1007/s12328‐020‐01179‐4
11	Yagnik, 2020	39/M	5.0 × 2.9	Terminal ileum	Surgery	10.1055/s‐0040‐1710531
12	Yu et al., 2017	15/F	11.5 × 9.0	Small intestine	Surgery	10.5152/tjg.2016.0513
13	Amouei et al., 2016	5/M	10.0	Ileum	Surgery	10.1016/j.ijscr.2016.03.025
14	Kiziltan et al., 2016	38/M	4.0 × 3.0	Ileum	Surgery	10.12669/pjms.321.9326
15	Dulskas et al., 2016	42/F	3.0	Jejunum (multiple)	Surgery	10.3892/ol.2015.4060
16	Unver et al., 2015	27/F	5.5	Ileum	Surgery	10.1016/j.amsu.2015.07.001
17	Present case, 2026	24/F	2.2 × 2.0	Ileum	Surgery	—

Preoperative endoscopic descriptions of primary small bowel IMT are rare, limited to two prior reports: an erythematous SMT‐like lesion seen via single‐balloon enteroscopy [7], and an intussuscepted tumor viewed during colonoscopy [8]. Our case uniquely visualized a deep small bowel lesion in situ via CE, showing an erythematous SMT‐like protrusion with conspicuous whitish villi. We hypothesized that the erythema and villi reflected superficial inflammation and lymphatic congestion, respectively. Supporting this endoscopic interpretation, histopathological re‐evaluation revealed localized lymphatic dilation in the involved mucosa, which may partly correspond to the macroscopic whitish villi, even in the absence of severe edema.

While balloon‐assisted enteroscopy is the standard, unreachable lesions pose challenges. Although CT effectively identified the mass, it lacked mucosal detail. Here, CE provided crucial additive information by visualizing the atypical surface features of the SMT‐like lesion. Because standard endoscopic biopsy of such submucosal lesions is frequently non‐diagnostic, CE served as a key complementary modality to raise clinical suspicion and justify definitive surgical resection. CE carries a retention risk, particularly in patients with suspected tumors or obstructive histories. Thus, prior patency evaluation using a patency capsule, as in our case, is essential. Complete resection is the mainstay of treatment for IMT; however, recurrence can occur, and long‐term follow‐up is recommended [9]. In a clinicopathologic series, distant metastases occurred only in ALK‐negative IMTs, suggesting ALK positivity may indicate a lower metastatic risk [10].

Nevertheless, local recurrence can occur even after complete resection, underscoring the need for careful long‐term surveillance [9, 10]. Because IMT is a rare entity, no standardized postoperative surveillance protocol has been established; however, long‐term follow‐up is widely recommended given the potential for recurrence [9, 10]. Previous reports have commonly described follow‐up imaging at intervals of approximately six months [6]. In the present case, complete surgical resection was achieved, and postoperative surveillance with imaging every six months has been continued. The patient remains disease‐free at the time of this report. However, this relatively short follow‐up is a limitation given the intermediate‐grade nature of IMT. Thus, long‐term cross‐sectional imaging surveillance will be continued.

In conclusion, we report a rare case of primary small bowel ALK‐positive IMT with characteristic findings on CE. Capsule endoscopy may serve as a valuable complementary modality for detecting subtle mucosal abnormalities when cross‐sectional imaging cannot fully characterize a lesion. IMT should be included in the differential diagnosis of erythematous small bowel lesions, particularly in young patients.

## Author Contributions

**Tomoyuki Niwa**: conceptualization, data acquisition, and writing – original draft. **Yasushi Hamaya**: conceptualization, supervision, and writing – review and editing. **Yusuke Asai**, **Tatsuhiro Ito**, **Satoru Takahashi**, **Shunya Onoue**, and **Satoshi Osawa**: writing – review and editing. **Mayu Sakata**: writing – review and editing, investigation, resources, and project administration. **Hiroya Takeuchi**: Writing – review and editing, investigation, resources. **Ken Sugimoto**: Supervision and writing – review and editing.

## Funding

The authors have nothing to report.

## Ethics Statement

According to institutional policy, ethical review and approval were not required for this case report.

## Conflicts of Interest

The authors declare no conflicts of interest.

## Consent

Written informed consent was obtained from the patient for publication of this case report and accompanying images.

4

## Data Availability

The data that support the findings of this study are available from the corresponding author upon reasonable request.
