# Correction to “Primary Anaplastic Lymphoma Kinase‐Positive Inflammatory Myofibroblastic Tumor of the Small Bowel Detected by Capsule Endoscopy: A Case Report”

**DOI:** 10.1002/deo2.70462

**Published:** 2026-11-04

Niwa T, Hamaya Y, Asai Y, Ito T, Takahashi S, Onoue S, Osawa S, Sakata M, Takeuchi H, Sugimoto K. Primary Anaplastic Lymphoma Kinase‐Positive Inflammatory Myofibroblastic Tumor of the Small Bowel Detected by Capsule Endoscopy: A Case Report. *DEN Open*. 2026;6(1):e70327. https://doi.org/10.1002/deo2.70327

In the author list of the above article, the affiliation of the first author was omitted. The affiliation for the first author is as follows: Department of Endoscopic and Photodynamic Medicine, Hamamatsu University School of Medicine, Shizuoka, Japan. The online article is corrected.

We apologize for this error.

